# Supplementary material for: The fungal peptide toxin candidalysin induces distinct membrane repair mechanisms compared to bacterial pore-forming toxins
Source: Cell Death Discov. 2025 Dec 27;12:62. doi: 10.1038/s41420-025-02923-w (PMC12847973; doi:10.1038/s41420-025-02923-w)
Supplement: Supplementary file 1 — Supplement [file 41420_2025_2923_MOESM1_ESM.pdf]

The fungal peptide toxin candidalysin induces distinct membrane repair mechanisms compared to bacterial pore-forming toxins

Roshan Thapa, Victor Kayejo, Claire M. Lyon, Bernhard Hube, Julian R. Naglik and Peter A. Keyel

Supplemental Tables and Legends

Graphical Abstract. Candidalysin is resisted by distinct repair mechanisms compared to bacterial PFTs. After pore formation and membrane damage by each toxin, multiple repair pathways are triggered downstream of Ca^{2+} flux. Candidalysin induces a protective Cl^- influx and activates MEK-dependent repair, which contributes to cell protection. Annexin translocation occurs slowly and provides minor protection, while patch repair is ineffective. In contrast, aerolysin does not benefit from Cl^- influx or MEK protection. Aerolysin triggers moderate annexin translocation and relies primarily on patch repair as the main protective mechanism. Streptolysin O elicits rapid annexin translocation and activates MEK signaling, both of which contribute to robust protection. Patch repair plays only a minor protective role against SLO. The figure was created using BioRender.

Supplemental Tables

Supplementary Table S1. Lytic parameters of toxins used

**Toxin**	**Figure Used**	**Hemolytic Activity (HU/mL)**	**Protein Concentration (mg/mL)**	**Specific Activity (HU/mg)**
Candidalysin WT	1A, D, E, 2A-C, 3A-E, 4A, B, 5C-I, 6A, S1A, S2A, S3A-D, S4A-E, S5A, S6A, S7A-C	5 $\times 10^4$	10	5 $\times 10^3$
Candidalysin^{AA}	1A, 5D, G, 6A, S7A-C	5 $\times 10^3$	10	5 $\times 10^2$
Aerolysin WT	1B, D, 2A, B, 3A, 4A, C, 5C, D-I, 6A, S3B, C, S4A, S5B, S6B, S7A-C	4 $\times 10^5$	2.8	1.43 $\times 10^5$
	1E, 2C, 3B-E, 7A, S1B, S2B, S3A, D, S4B-E,	6.7 $\times 10^5$	2.4	2.8 $\times 10^5$
Aerolysin^{Y221G}	1B	<1 $\times 10^3$	1.6	<6.25 $\times 10^2$
SLO WT	1C-E, 3A, S1C, S2C, S3A, S4A	6 $\times 10^5$	1.5	4 $\times 10^5$
	2B, 3B, C, 4D, 5C-I, 6A, S3B, S4B, C, S5C, S6C, S7A-C	1 $\times 10^6$	2.4	4.2 $\times 10^5$
	2A, C, D, 3B-E, 4A, 5C, S3B, D, S4B-E, S6C	2 $\times 10^6$	5.3	3.8 $\times 10^5$
SLO ML	1C	<1 $\times 10^3$	3.2	<3.13 $\times 10^2$

Supplemental Figure Legends

Supplementary Figure S1. Normalization of toxin activity using hemolytic activity. Human red blood cells were washed and diluted to 2% in RBC Assay buffer. Then they were challenged with serially diluted toxins, incubated at 37°C for 30 min and centrifuged to pellet remaining RBCs. The A_{450} of the supernatants were determined. One hemolytic unit is defined as the amount of toxin that is required to lyse 50% of a 2% human RBC solution in 30 min at 37°C. The graph displays one representative RBC lysis titration from at least 3 independent experiments.

Supplementary Figure S2. Cytolytic activity of Candidalysin plateaus at 1 h. HeLa cells were challenged with (A) candidalysin (Clys), (B) aerolysin (Aero) or (C) streptolysin O (SLO) at the indicated concentrations in phenol-red free DMEM supplemented with 2 mM $CaCl_2$ for 30 min, 1 h and 2 h. Viability of cells was assessed by MTT assay and % specific lysis was determined. The LC_{50} was calculated as described in the methods. Graphs display mean ± S.E.M of 3 independent experiments, with individual points representing independent experiments. The dotted lines indicate the limits of detection. * $p < 0.05$, ns not significant by repeated-measures ANOVA with Tukey post-test.

Supplementary Figure S3. LDH assay under-reports killing by toxins. HeLa cells were challenged with (A) candidalysin (Clys), (B) aerolysin (Aero) and (C) SLO at the indicated concentrations in phenol-red free DMEM supplemented with 2 mM $CaCl_2$ for 30 min, 1 h and 2 h. Cells were centrifuged at 1200xg for 5 min and cell pellet and supernatant were separated. The % specific lysis and raw cytotoxicity was determined by LDH assay. The graphs display the mean ± S.E.M of 3 independent experiments. * $p < 0.05$, ** $p < 0.01$ by repeated-measures ANOVA with Tukey post-test.

Supplementary Figure S4. Ca^{2+}-dependent repair is essential for cellular protection against candidalysin but not patch repair and ceramide. (A) HeLa cells or (B) C2C12 control and *Dysf* shRNA cells were challenged with the indicated concentrations of candidalysin (Clys), aerolysin (Aero) or SLO in RPMI with 20 µg/ml PI supplemented with either 2 mM $CaCl_2$ or (A) 2 mM EGTA. (C) HeLa cells were either untransfected, or transfected with GFP or GFP Dysferlin (GFP-Dysf) for 48 h and challenged with 9.375-600 HU/mL of Clys, or 31-2000 HU/mL of Aero for 1 h or challenged with the indicated concentrations of Clys, Aero or SLO. (D) HeLa cells were pretreated with either DMSO (CTL) or 40 µM PPMP for 72 h. Then cells were challenged with the indicated concentrations of Clys, Aero or SLO. PI uptake was analyzed by flow cytometry and specific lysis was determined. Graphs show the mean ± S.E.M of (A) 5 or (B-D) 4 independent experiments.

Supplementary Figure S5. Chloride ions play a crucial role in protecting cells from candidalysin. (A) HeLa cells were challenged with the indicated concentrations of candidalysin (Clys), aerolysin (Aero), or SLO in RPMI supplemented with or without 150 mM KCl. (B) Caspase $1/11^{-/-}$ bone-marrow derived macrophages were challenged with the indicated concentrations of Clys, Aero or SLO in RPMI supplemented with 2 mM $CaCl_2$ or 2 mM EGTA and/or 150 mM KCl. (C) HeLa cells were challenged with Clys at indicated concentrations in RPMI supplemented with the indicated combinations of 2 mM $CaCl_2$, 2 mM EGTA, 20 µM nigericin or 150 mM KCl. (D, E) HeLa cells were challenged with the indicated concentrations of Clys, Aero or SLO in RPMI with 2 mM $CaCl_2$ supplemented with nothing (Ca^{2+}), 300 mM dextrose, 150 mM NaCl, 150 mM KCl, 150 mM potassium acetate, or 150 mM NH_4Cl. (F) HeLa or A431 vaginal epithelioid cells were challenged with Clys, Aero, or SLO at the indicated concentrations in RPMI supplemented with 2 mM $CaCl_2$. (G) A431 cells were challenged with the indicated concentrations of Clys in RPMI supplemented with 2 mM $CaCl_2$ and either: nothing, 150 mM NaCl, 150 mM KCl, 300 mM dextrose, 150 mM potassium acetate, or 150 mM

NH_4Cl. PI uptake was analyzed by flow cytometry and specific lysis was determined. Graphs show the mean ± S.E.M. of 3 (A, C, F: Aero), 4 (B, D, G), or 5 (F:Clys and SLO) 5 independent experiments.

Supplementary Figure S6. Ca^{2+}-mediated MEK activation promotes limited cellular resistance to candidalysin. HeLa cells were serum starved for 30 min and pre-treated with 20 μM MEK inhibitor U0126 or vehicle DMSO for 30 min. Cells were challenged in RPMI supplemented either with 2 mM $CaCl_2$ or 2 mM EGTA using the indicated concentrations of (A) candidalysin (Clys), (B) aerolysin (Aero), or (C) SLO. PI uptake was analyzed by flow cytometry and specific lysis was determined. Graphs show the mean ± S.E.M of 4 independent experiments.

Supplementary Figure S7. Transient depletion of annexins sensitizes cells to candidalysin. HeLa cells were transfected with siRNAs to control (CTL) or annexins for 72 h. Cells were then challenged with indicated concentrations of (A) candidalysin (Clys), (B) aerolysin (Aero), or (C) SLO. PI uptake was analyzed by flow cytometry and specific lysis was determined. Graphs show the mean ± SEM of (A-C) 3 independent experiments.

Supplementary Figure S8. Delayed translocation of annexins occurs following candidalysin challenge. HeLa cells transfected with A2-GFP or A6-YFP were challenged with sublytic toxin concentrations (50 HU/mL candidalysin (Clys), 62 HU/mL aerolysin (Aero) or 250 HU/mL SLO) or a mass equivalent of candidalysin^{AA} (Clys^{mutAA}). The cells were imaged by confocal microscopy for ~45 min at 37°C and then lysed with 1% Triton-X-100. The mean ± SEM of (A) annexin depletion from the cytosol, (B) annexin enrichment on the membrane, or (C) TO-PRO3 uptake over time is graphed normalized to maximal intensity. For each toxin, 25–30 cells from 3 independent experiments were analyzed. Arrowheads indicate toxin addition or Triton addition.

Supplementary Figure S9. Original Western blots for Fig 5. Uncropped blots from Fig 5 are shown.

Supplementary Figure S10. Original Western blots for Fig 6. Uncropped blots from Fig 6 are shown.

Supplementary Figure S11. Original Western blots for Fig 7A. Uncropped blots from Fig 7A are shown.

Supplementary Figure S12. Original Western blots for Fig 7B. Uncropped blots from Fig 7B are shown.

Supplemental Video Legends

Video V1. Cells utilize annexin A6 mediated microvesicle shedding to remove candidalysin pores. HeLa cells transfected with annexin A6-YFP (green) were challenged with sublytic (A) candidalysin (CLY WT), (B) candidalysin^{AA} (CLY mutAA), (C) aerolysin (Aero), or (D) SLO in the presence of 2 μg/mL TO-PRO3 (blue) and imaged at 37°C by live cell confocal imaging at 1-3.5 frame/second. Triton-X-100 was added at the end of 45 min of imaging as a positive control for cell permeabilization. Images were bleach-corrected by histogram matching. Time shows min after toxin addition. Scale bar = 10 μm.

Video V2. Candidalysin triggers delayed annexin A2 translocation to membrane. HeLa cells transfected with annexin A2-GFP (green) were challenged with sublytic (A) candidalysin (CLY WT), (B) candidalysin^{AA} (CLY mutAA), (C) aerolysin (Aero), or (D) SLO in the presence of 2 μg/mL TO-PRO3 (blue) and imaged at 37°C by live cell confocal imaging at 1-3.5 frame/second. Triton-X-100 was added at the end of 45 min of imaging as a positive control for cell permeabilization. Images were bleach-corrected by histogram matching. Time shows min after toxin addition. Scale bar = 10 μm.

Figure S1

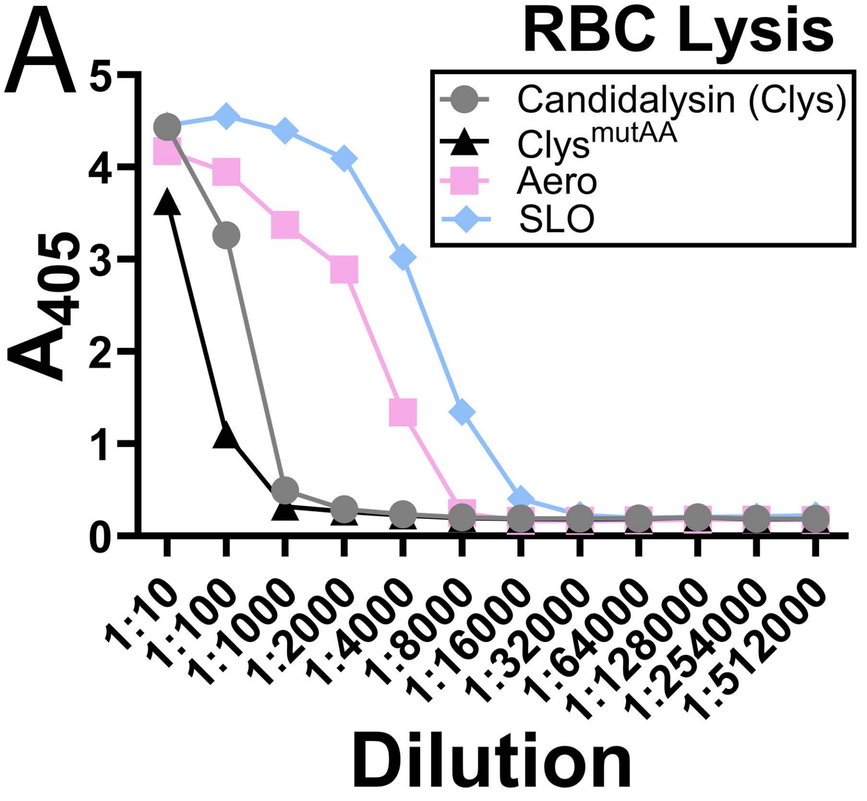

A
RBC Lysis
Candidalysin (Clys)
Clys mutAA
Aero
SLO
A405
0
1
2
3
4
5
1:10
1:100
1:1000
1:2000
1:4000
1:8000
1:16000
1:32000
1:64000
1:128000
1:254000
1:512000
Dilution

Figure S2

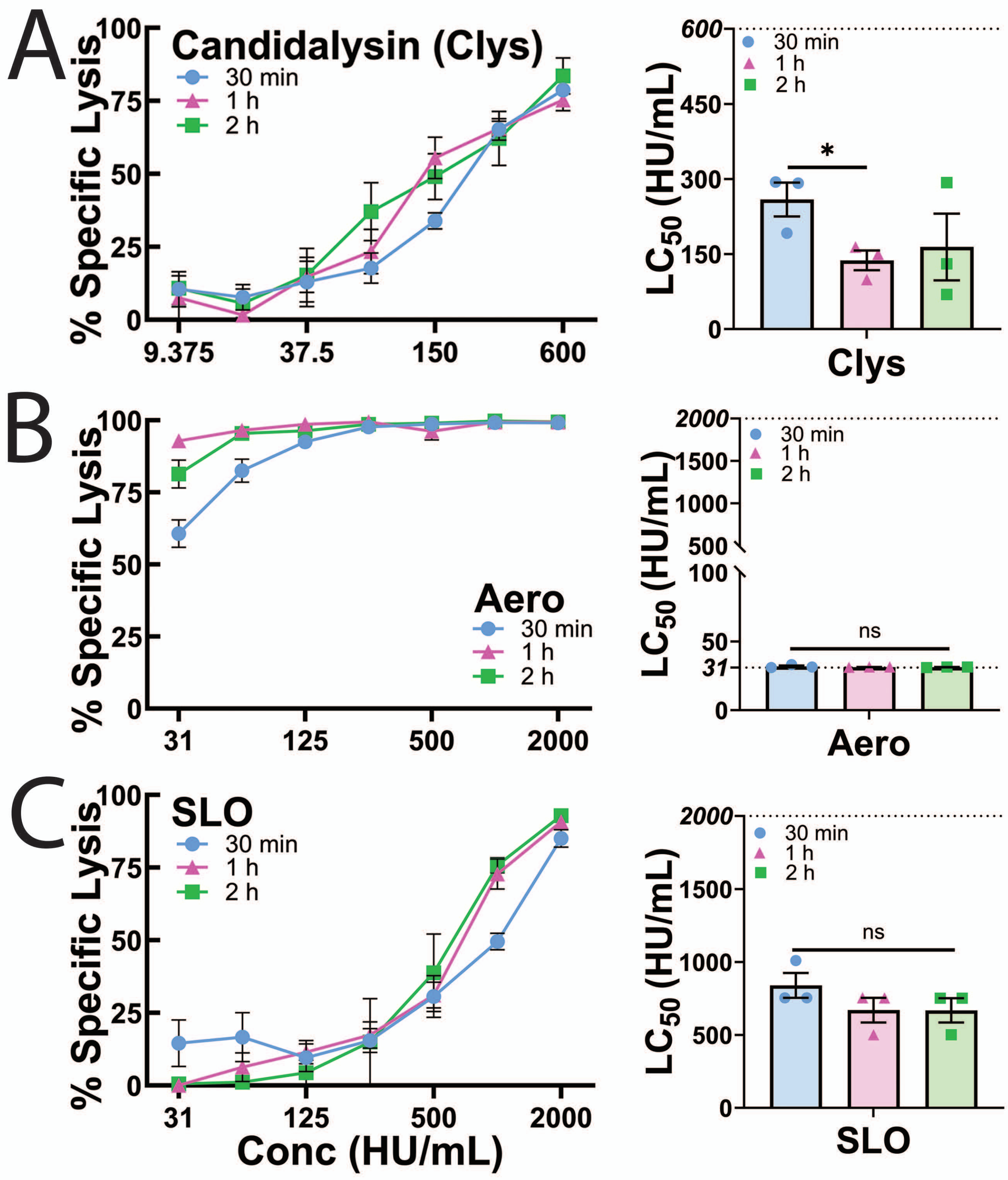
A
Candidalysin (Clys)
% Specific Lysis
100
75
50
25
0
9.375
37.5
150
600
30 min
1 h
2 h
LC50 (HU/mL)
600
450
300
150
0
*
Clys
B
% Specific Lysis
Aero
30 min
1 h
2 h
31
125
500
2000
2000
1500
1000
500
100
50
31
ns
Aero
C
SLO
% Specific Lysis
Conc (HU/mL)
31
125
500
2000
LC50 (HU/mL)
2000
1500
1000
500
0
ns
SLO

Figure S3

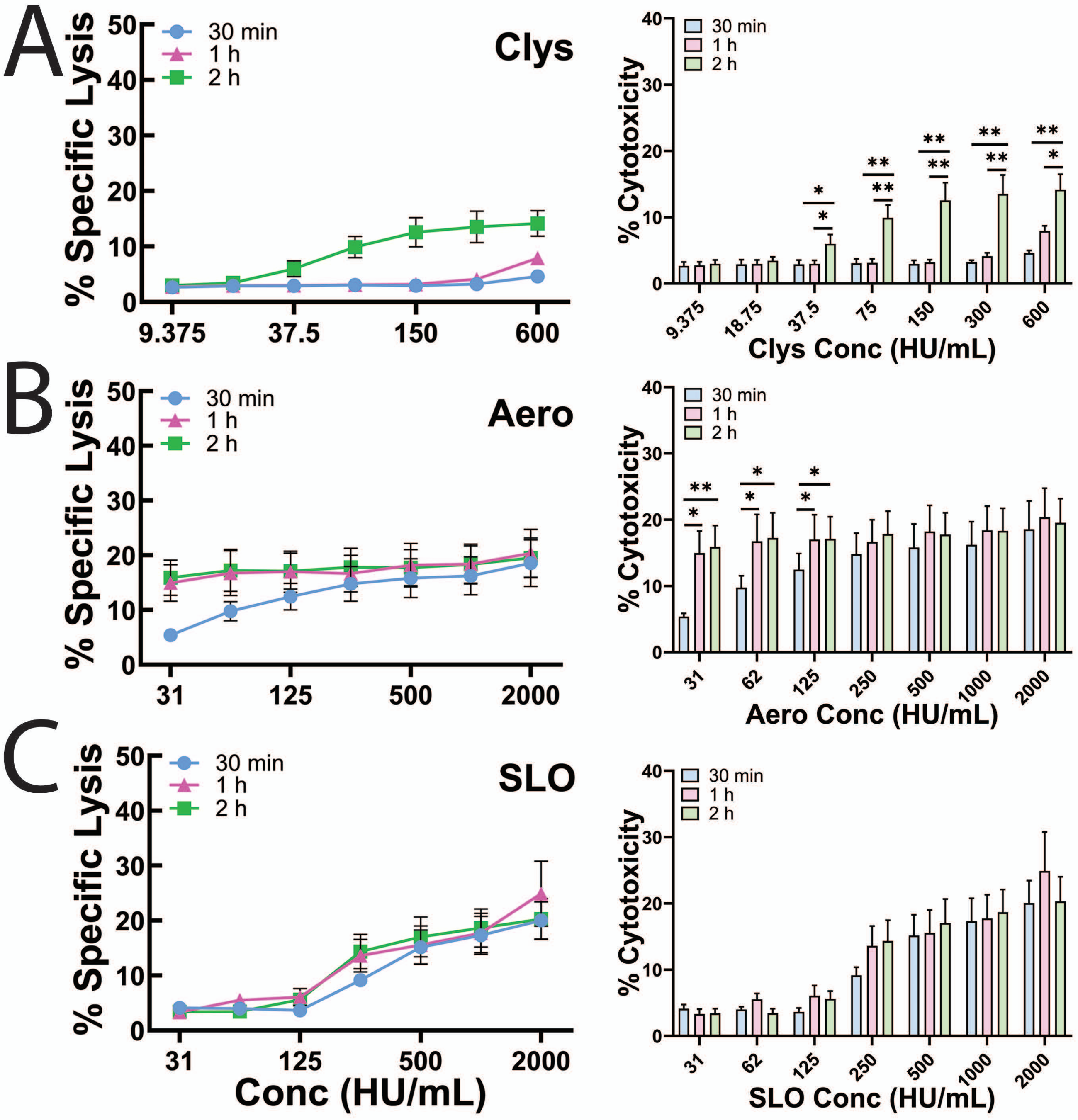
A
B
C
Clys
Aero
SLO
% Specific Lysis
% Cytotoxicity
30 min
1 h
2 h
Clys Conc (HU/mL)
Aero Conc (HU/mL)
SLO Conc (HU/mL)
Conc (HU/mL)

Figure S4

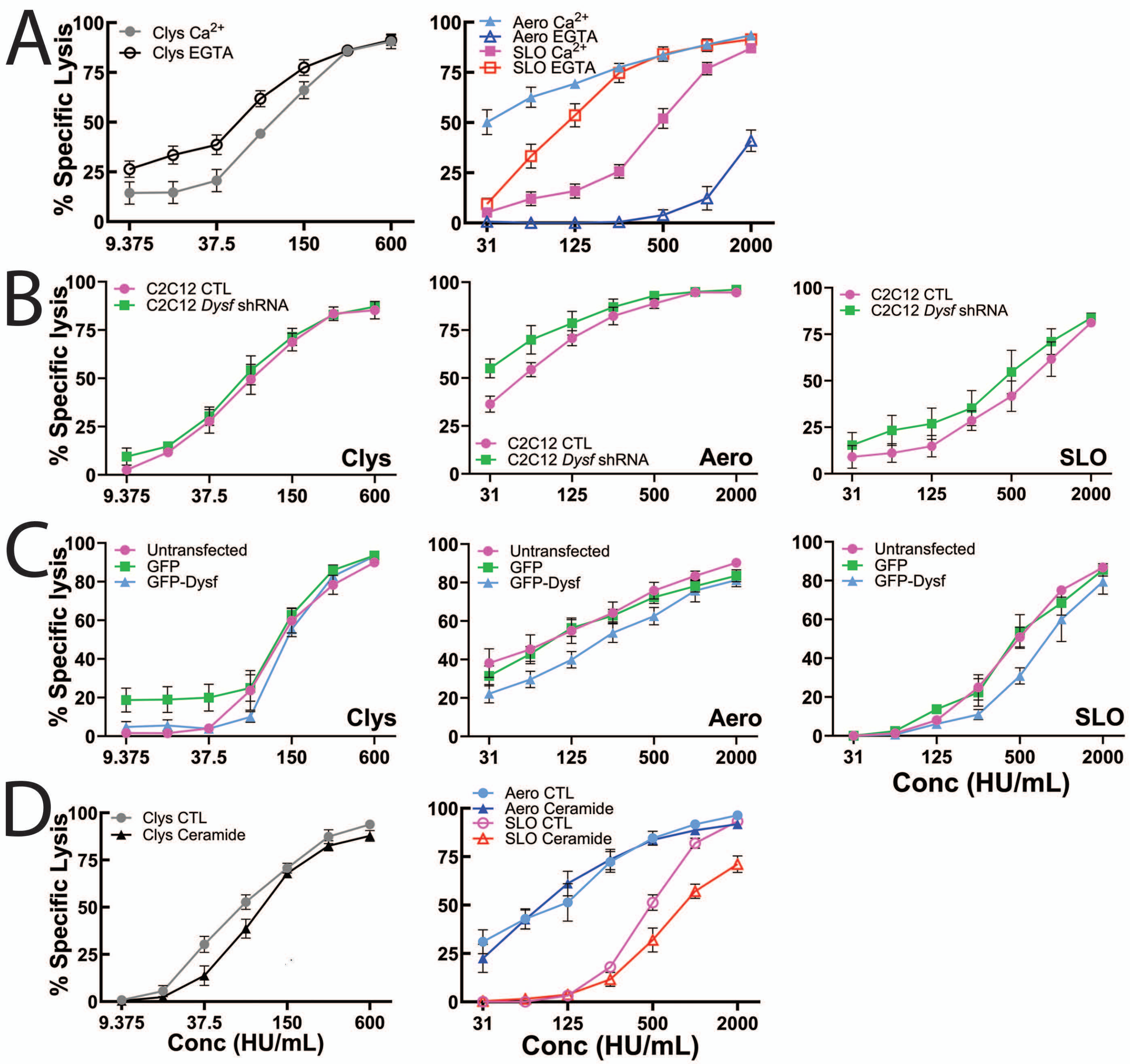

A
% Specific Lysis
Clys Ca2+
Clys EGTA
Aero Ca2+
Aero EGTA
SLO Ca2+
SLO EGTA
B
% Specific lysis
C2C12 CTL
C2C12 Dysf shRNA
Clys
Aero
SLO
C
Untransfected
GFP
GFP-Dysf
Conc (HU/mL)
D
Clys CTL
Clys Ceramide
Aero CTL
Aero Ceramide
SLO CTL
SLO Ceramide

Figure S5

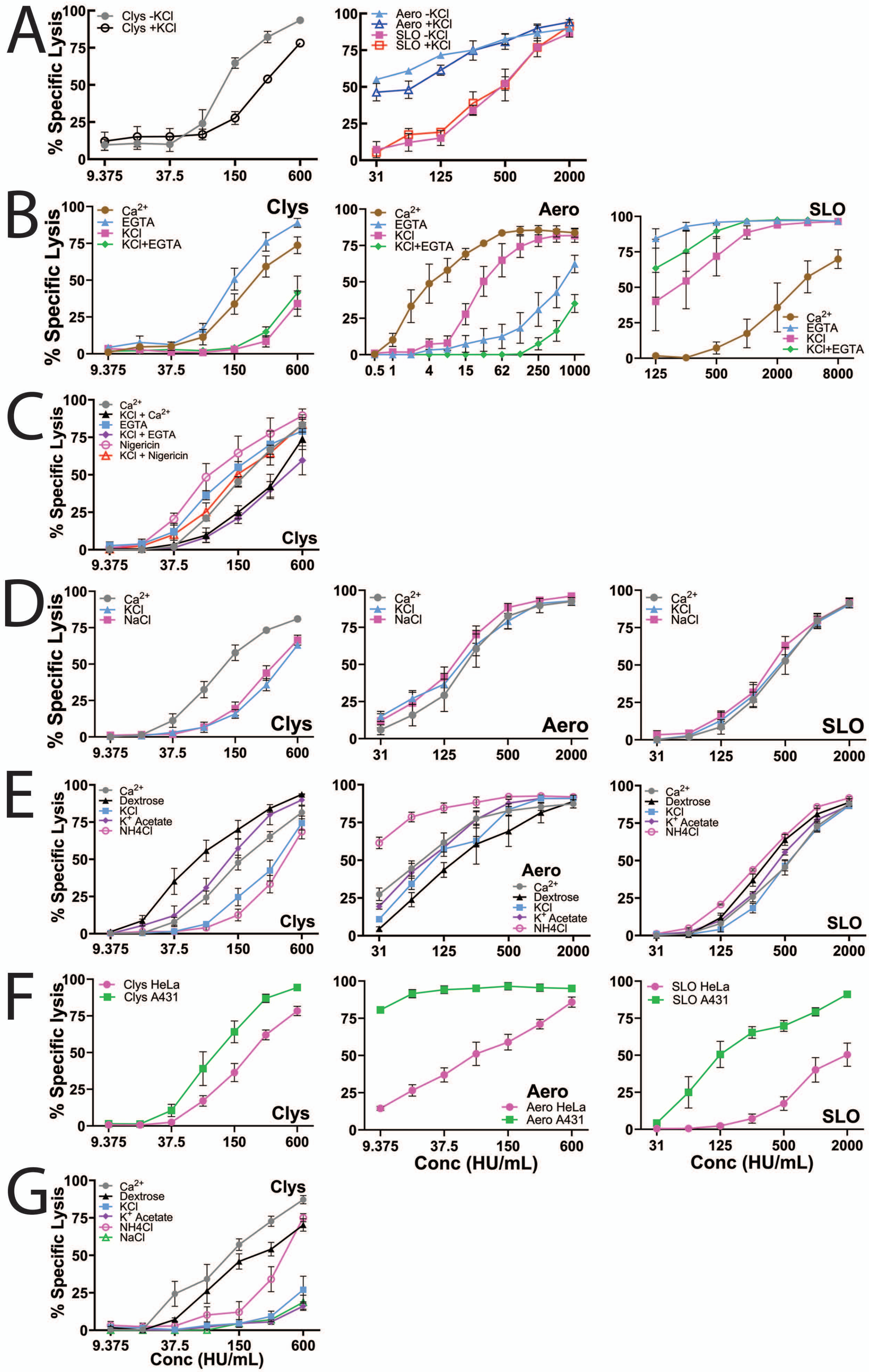
A
% Specific Lysis
Clys -KCl
Clys +KCl
Aero -KCl
Aero +KCl
SLO -KCl
SLO +KCl
B
Clys
Aero
SLO
Ca2+
EGTA
KCl
KCl+EGTA
C
Ca2+
KCl + Ca2+
EGTA
KCl + EGTA
Nigericin
KCl + Nigericin
Clys
D
Ca2+
KCl
NaCl
Clys
Aero
SLO
E
Ca2+
Dextrose
KCl
K+ Acetate
NH4Cl
Clys
Aero
SLO
F
% Specific lysis
Clys HeLa
Clys A431
Clys
Aero
Aero HeLa
Aero A431
SLO HeLa
SLO A431
SLO
Conc (HU/mL)
G
Ca2+
Dextrose
KCl
K+ Acetate
NH4Cl
NaCl
Clys
Conc (HU/mL)

Figure S6

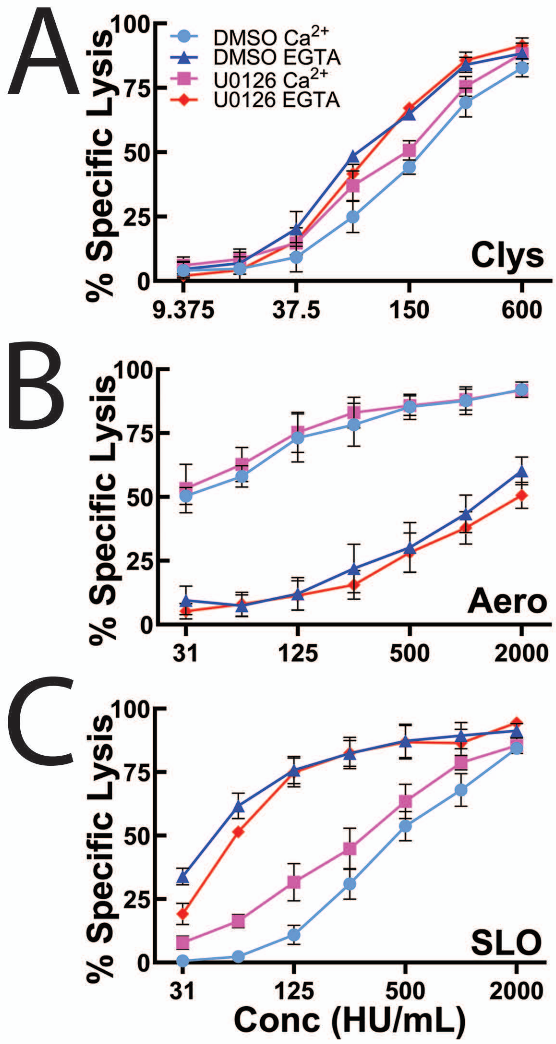
A
DMSO Ca2+
DMSO EGTA
U0126 Ca2+
U0126 EGTA
% Specific Lysis
100
75
50
25
0
Clys
9.375
37.5
150
600
B
% Specific Lysis
100
75
50
25
0
Aero
31
125
500
2000
C
% Specific Lysis
100
75
50
25
0
SLO
31
125
500
2000
Conc (HU/mL)

Figure S7

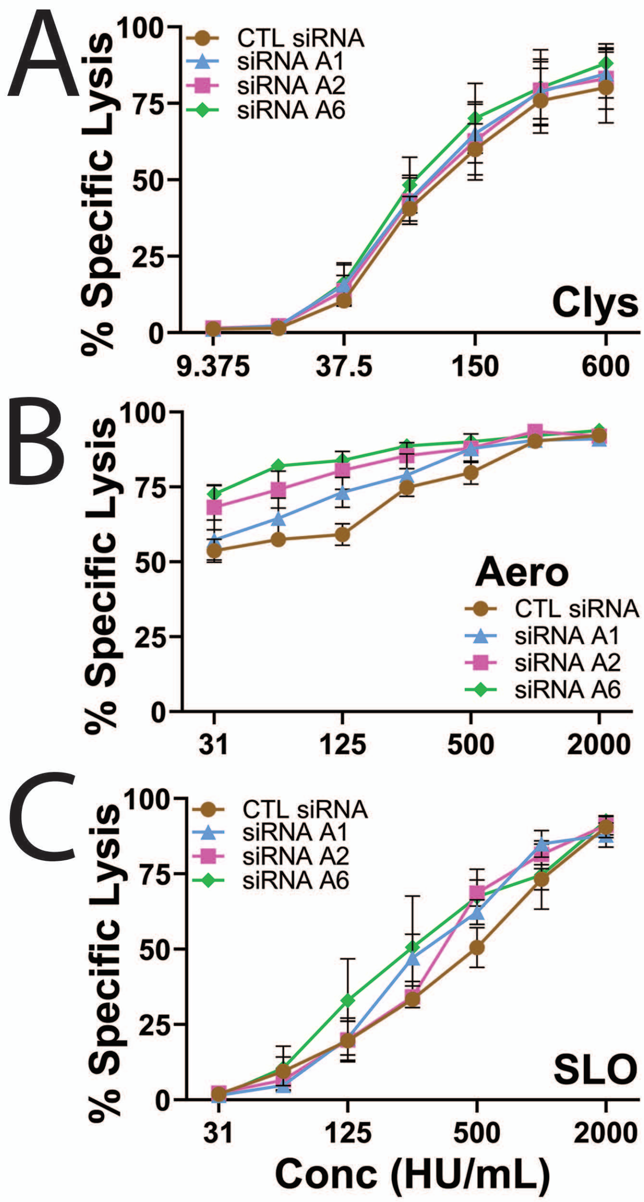
A
% Specific Lysis
100
75
50
25
0
CTL siRNA
siRNA A1
siRNA A2
siRNA A6
Clys
9.375
37.5
150
600
B
% Specific Lysis
100
75
50
25
0
Aero
CTL siRNA
siRNA A1
siRNA A2
siRNA A6
31
125
500
2000
C
% Specific Lysis
100
75
50
25
0
CTL siRNA
siRNA A1
siRNA A2
siRNA A6
SLO
31
125
500
2000
Conc (HU/mL)

Figure S8

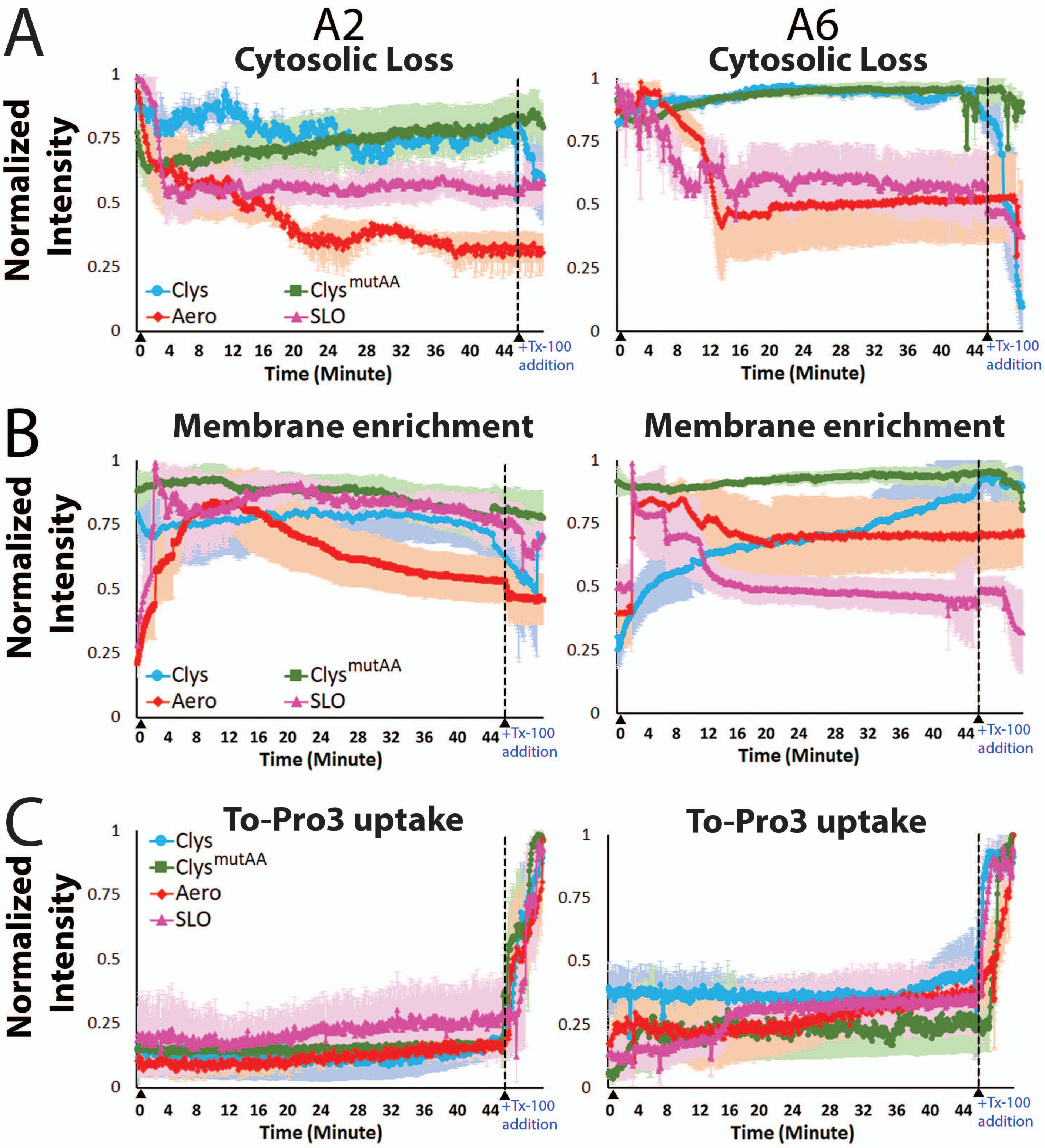

A
A2
Cytosolic Loss
A6
Cytosolic Loss
Normalized Intensity
1
0.75
0.5
0.25
0
Clys
Clys mutAA
Aero
SLO
0 4 8 12 16 20 24 28 32 36 40 44
+Tx-100 addition
Time (Minute)
B
Membrane enrichment
Membrane enrichment
C
To-Pro3 uptake
To-Pro3 uptake
