# Supplementary figures and images for: The fungal peptide toxin candidalysin induces distinct membrane repair mechanisms compared to bacterial pore-forming toxins
Source: Cell Death Discov. 2025 Dec 27;12:62. doi: 10.1038/s41420-025-02923-w (PMC12847973; doi:10.1038/s41420-025-02923-w)

Figure S9

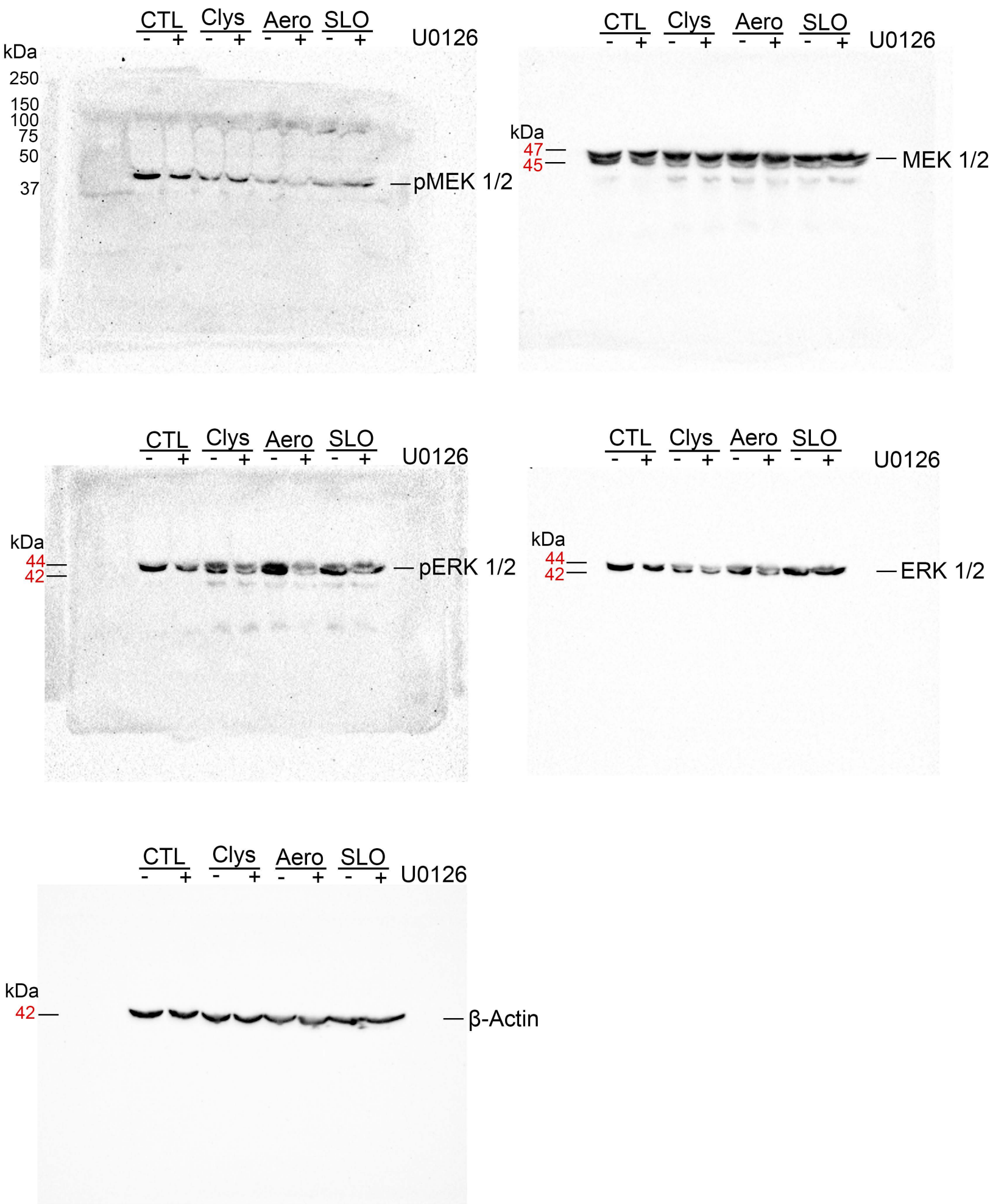

Figure S10

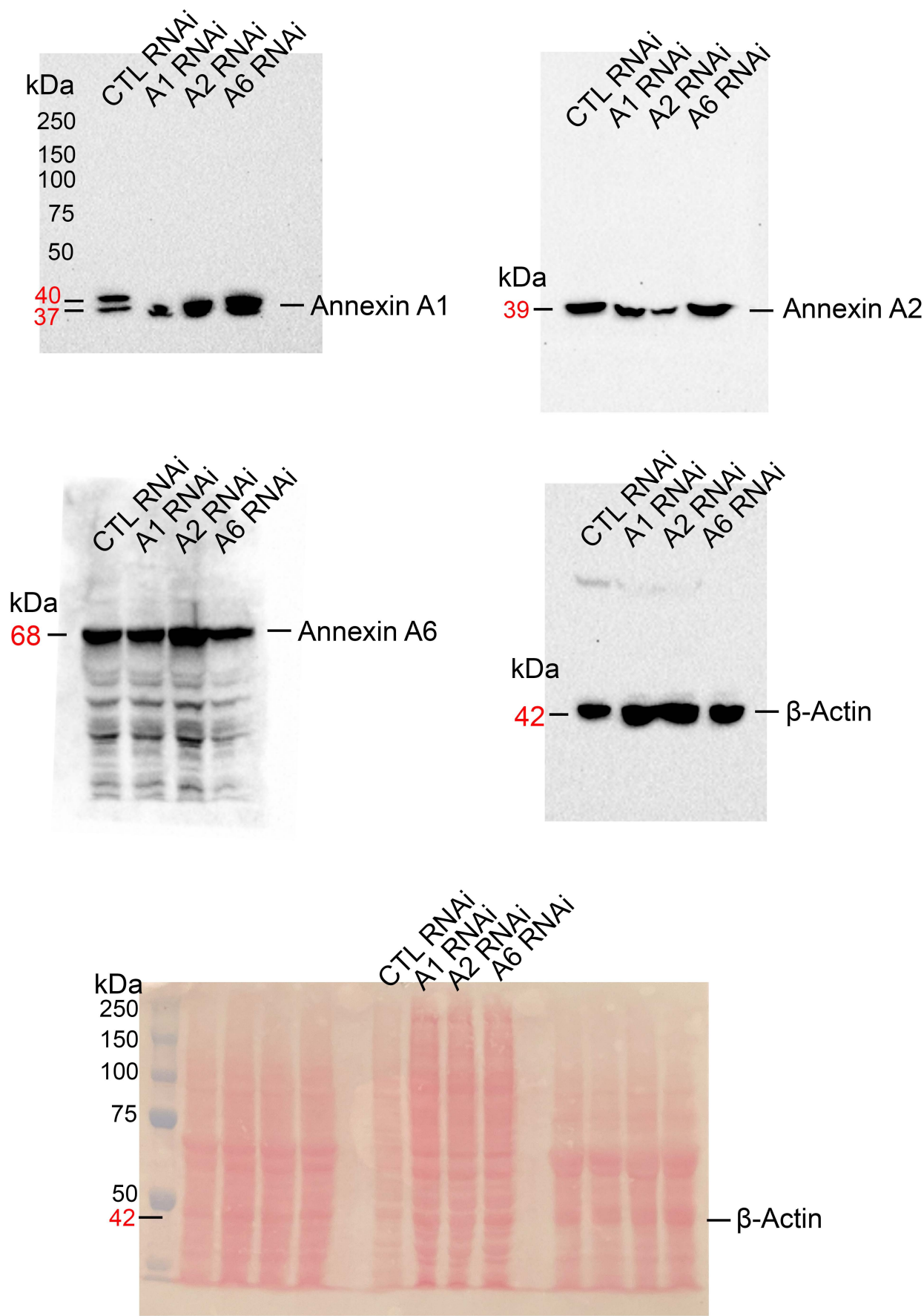

Ponceau stain of the blots

# Figure S11

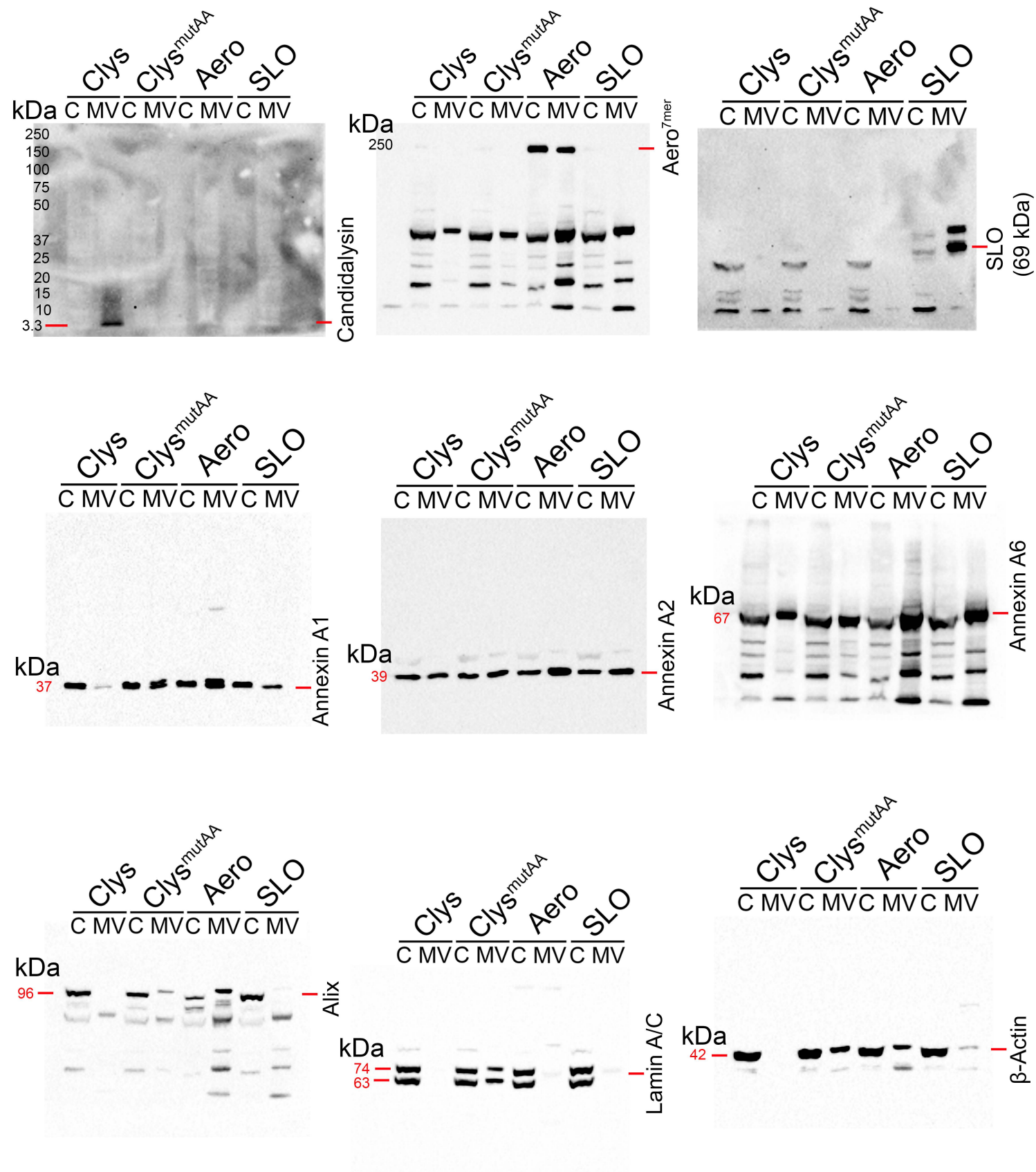

# Figure S12

## Dot Blot Assay

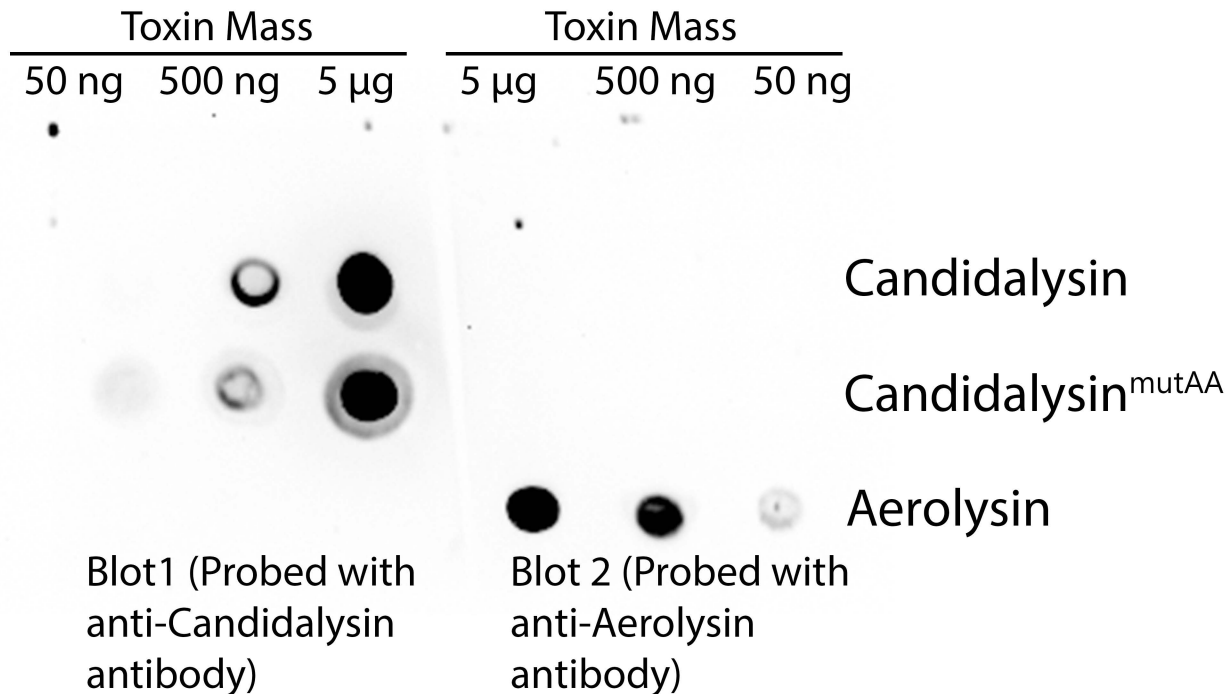

Supplement: Supplementary file 2 — uncropped western blots [file 41420_2025_2923_MOESM2_ESM.pdf]
